# Magnetism of Nanoparticles: Effect of the Organic Coating

**DOI:** 10.3390/nano11071787

**Published:** 2021-07-09

**Authors:** Maryam Abdolrahimi, Marianna Vasilakaki, Sawssen Slimani, Nikolaos Ntallis, Gaspare Varvaro, Sara Laureti, Carlo Meneghini, Kalliopi N. Trohidou, Dino Fiorani, Davide Peddis

**Affiliations:** 1 Istituto di Struttura della Materia—CNR, Monterotondo Scalo, 00015 Rome, Italy; maryam.abdolrahimi@uniroma3.it (M.A.); Sawssen.Slimani@edu.unige.it (S.S.); gaspare.varvaro@ism.cnr.it (G.V.); sara.laureti@ism.cnr.it (S.L.); dino.fiorani@ism.cnr.it (D.F.); 2 Dipartimento di Scienze, Università degli Studi Roma Tre, 00146 Rome, Italy; carlo.meneghini@uniroma3.it; 3 Institute of Nanoscience and Nanotechnology, NCSR “Demokritos”, 15310 Agia Paraskevi, Attiki, Greece; m.vasilakaki@inn.demokritos.gr (M.V.); n.ntallis@inn.demokritos.gr (N.N.); k.trohidou@inn.demokritos.gr (K.N.T.); 4 Laboratory of Applied Physics, Faculty of Sciences of Sfax, University of Sfax, B.P. 1171, Sfax 3000, Tunisia; 5 Dipartimento di Chimica e Chimica Industriale, Università degli Studi di Genova, Via Dodecaneso 31, 16146 Genova, Italy

**Keywords:** magnetic nanoparticles, molecular coating, magnetic properties

## Abstract

The design of novel multifunctional materials based on nanoparticles requires tuning of their magnetic properties, which are strongly dependent on the surface structure. The organic coating represents a unique tool to significantly modify the surface structure trough the bonds between the ligands of the organic molecule and the surface metal atoms. This work presents a critical overview of the effects of the organic coating on the magnetic properties of nanoparticles trough a selection of papers focused on different approaches to control the surface structure and the morphology of nanoparticles’ assemblies.

## 1. Introduction

Since the pioneering works of Louis Néel in 1944 [1], magnetic nanoparticles have been the subject of continuous interes [2,3,4,5,6,7,8,9], due to the possibilities of designing new magnetic systems with finely tuned properties, thanks to the enormous progress in the synthesis and diagnostic methods, and the increasing number of applications, e.g., biomedicine [10,11,12,13], ferrofluid technology [14], catalysis [15,16], sensors [17,18,19], magnetic energy storage [20,21], electronics [22] etc. Therefore, the understanding of the physics of magnetic nanoparticles and the control of their properties still represent a very attractive and challenging topic for both fundamental research and technological applications.

Entering the nanometer-scale regime (<100 nm), the magnetic properties of materials change progressively with decreasing size until they show substantial differences with respect to the bulk state when the fraction of atoms lying on the surface becomes larger than that in the particle core, making the role of the surface dominant. For this reason, in the last decades ever-increasing interest has been devoted to approaches aimed at controlling the contribution of the surface to optimize the magnetic properties for meeting the requirements for specific applications. 

The surface structure is disordered, due to the breaking of lattice symmetry, with random missing bonds and then incomplete atomic coordination sphere with respect to the atoms in the core and in the bulk state. This induces significant changes in the magnetic properties of nanoparticles, which become more and more important with decreasing particle size, i.e., increasing surface/volume ratio:

− increase of magnetic anisotropy due to the surface contribution arising from the random spin pinning on the surface. The “surface anisotropy” was described by Néel [23], using a phenomenological model which assumes the lack of atomic bonds on the surface of a crystal at the origin of this form of anisotropy. The surface anisotropy contribution was described as a pair interaction of nearest neighbors spins, depending on their distance and orientation. Later studies clarified the mechanism of the surface anisotropy pointing out the very important role of spin-orbit coupling and ligand field [24,25].
− decrease of saturation magnetization in ultrasmall particles because of the surface spin disorder that makes the surface magnetically inactive (dead layer). This represents a strong limitation for those applications of nanoparticles requiring high particle moments.

**Figure 1:**
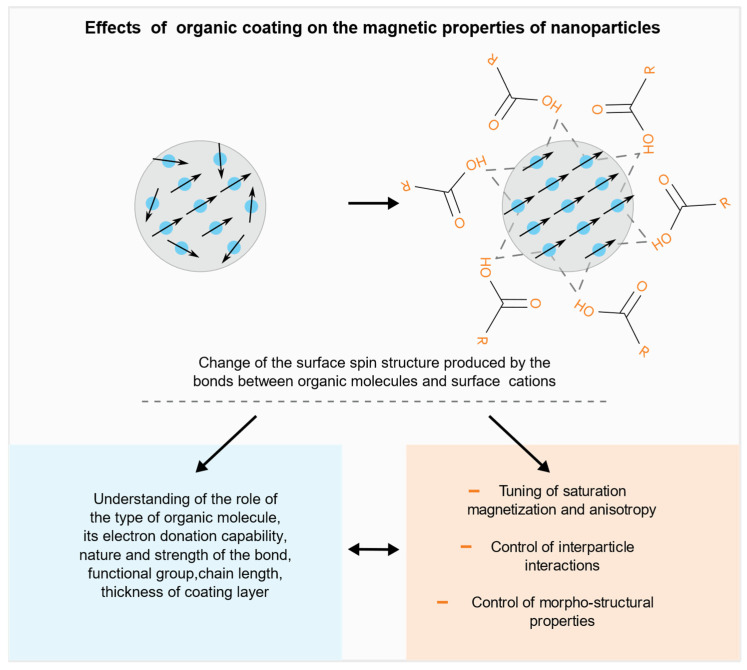
Schematic diagram showing the effect of the organic coating on the surface spin structure.

The surface [25,26] contribution can be controlled through the interaction with another phase in contact with it, i.e., exploiting the interface effect, which can be of different type depending on the nanoparticle system and the nature of the two phases in contact [27]. As an example, in bimagnetic core/shell particles, combining ferromagnetic and antiferromagnetic phases, the exchange coupling at the interface between the core and the surface shell can give rise to the exchange bias phenomenon [28,29], which is often accompanied by an increase of anisotropy, due to the so-called “exchange anisotropy”. The effect on the anisotropy and saturation magnetization can be tuned controlling the core diameter, the thickness of the surface and the chemical composition. The interface can play a different role when the phase in contact is non-magnetic, e.g., an organic molecule. It has been proved that the surfactant plays a strong role on the magnetic properties of the NPs surface reducing the disorder by replacing the missing bonds through proper ligands which bind to the surface restoring in such a way the bulk crystal environment (Figure 1).

The nanoparticles can be functionalized by a post synthesis process allowing to finely tune the magnetic properties of the single particle. The main effects of the reduction of the surface disorder produced by the organic coating on the magnetic properties are:

− increase of the particle saturation magnetization, due to the contribution of the surface, becoming magnetically active [30,31,32,33].
− decrease of the particle anisotropy, due to the decrease of the surface contribution [24,34].

A fine tuning of both saturation magnetization and anisotropy can be achieved through the control of the role of the factors that were found to significantly affect the above properties, such as the type of functional group of the ligands, their chain length, the nature and the strength of the bond (monodentate or bidentate), the packing density of the coating molecules, the electronic structure and surface disorder of the original particles, which depend on the synthesis route. It is worth pointing out that, due to the complexity and the number of the involved factors, in some cases contradictory results (e.g., decrease of the saturation magnetization) are observed, [35,36] in oxide particles with spinel structure. The organic coating can also produce other changes that have a direct impact on the magnetic properties of an assembly of nanoparticles, such as:

− controlled change of the interparticle distance, functionalizing the particles with molecules of proper steric features, allowing in such a way a tuning of the strength of dipolar interparticle interactions, which contribute to the effective anisotropy [37,38].
− controlled change, by specific ligands, of morpho-structural features of the particle assembly, i.e., size, shape and the crystalline structure of NPs during the synthesis process, strongly affecting the resulting magnetic state and its thermal stability [39,40,41].

Besides the effects on the magnetic properties, magnetic coating can provide new selective surface functionalities exploitable for several applications and can control the physical and chemical stability of nanoparticles avoiding aggregation and favoring their biocompatibility.

In this framework, the present paper is aimed at providing a critical overview, through a selection of publications on the different effects produced by the organic coating on the magnetic and morpho-structural properties of nanoparticle assemblies.

The paper is structured in sections focused on the following effects:

(a) Tuning of saturation magnetization and anisotropy
(b) Control of interparticle interactions
(c) Control of morpho-structural properties

## 2. Tuning of the Saturation Magnetization and Anisotropy

As previously reported, organic molecules can modify the surface structure through the ligands which actually replace the missing bonds, reducing the spin disorder, leading to a change of surface anisotropy [24,34] and saturation magnetization (M_S_) [30,31,32,33,41].

The mechanism of bonding to the nanoparticle surface depends on the type and strength of bonds as well as the geometry and electronic structure of the surface. Metal atoms located at the surfaces containing available d-bands can interact with small molecules having accessible π* states through Blyholder-type interaction [42]. Actually, a new surface can be built whose atomic environment can be predetermined choosing the suitable organic molecule with proper ligands able to bind to surface atoms.

Examples of different approaches to tune both saturation magnetization and anisotropy are reported below using different molecules with different functional groups of ligands. Advanced characterization techniques and theoretical models have been used to deeply investigate the interaction between molecules and surfaced atoms. Most evidence of the organic coating effects on the magnetic properties has been reported on oxide nanoparticles.

A detailed structural and theoretical investigation of the coordination symmetry of ligands and calculation of atomic distances on the surface were performed by Salafranca et al. [33]. That paper provides evidence of the changes in the surface electronic structure produced the coating and highlights the key role of the nature and number of organic molecules in the fundamental magnetic properties of nanoparticles. The authors used aberration-corrected scanning transmission electron microscopy (STEM), electron energy loss spectroscopy (EELS), electron magnetic chiral dichroism (EMCD) measurements, combined with density functional theory (DFT) calculations to map the magnetization of oleic acid coated magnetite nanoparticles (from 6 to 15 nm) in real space with sub-nanometer spatial resolution. DFT calculations reproduced well the measured O K-edge EELS fine structure and provided the most stable atomic configuration. It was found that the surface bonding with the acid’s oxygen atoms results in O-Fe atomic configuration and distances close to the bulk values. Indeed, the oxygen of the carboxylic group completes the octahedral environment of the bonding Fe atoms, making their first coordination like bulk magnetite. For the Fe ions bonded to the organic acid oxygens, the Fe-O average distance is 2.05 Å close to 2.10 Å in the bulk. Consequently, their densities of states occupancies are like those of bulk Fe_3_O_4_. For the half of the surface Fe ions that do not bond to the acid’s oxygen atoms, the number of O nearest neighbors is 5, as in the bare surface, and the average Fe-O distance is 1.92 Å, only 0.04 Å smaller than that at the bare surface.

**Figure 2:**
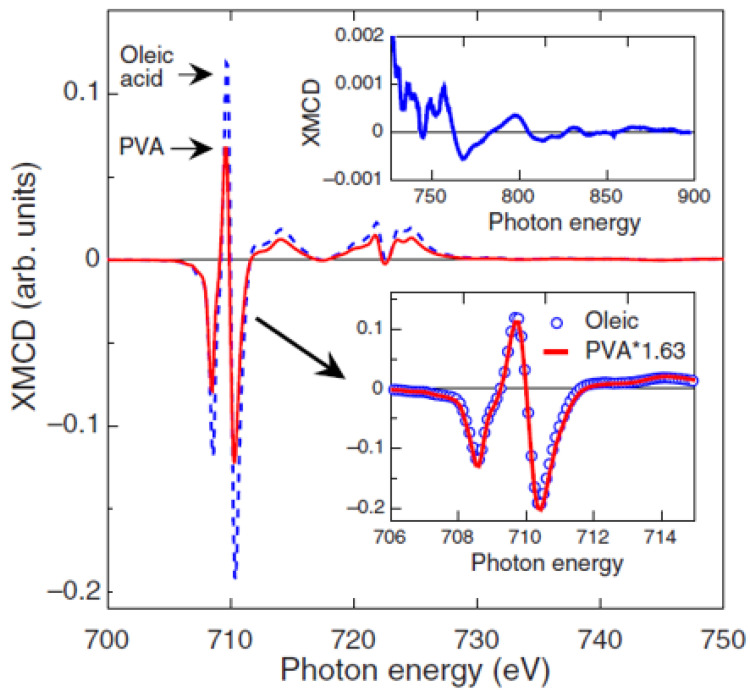
XMCD curves at the L_2,3_ Fe edges recorded on the oleic acid (dashed blue line) and PVA (solid red line) samples. Upper inset: detail of the high-energy XMCD curve of the oleic acid sample. Bottom inset: detail of the scaling of the XMCD curves at the Fe L_3_ edge measured on both samples. The Figure is reprinted with permission from Ṕrez et al. [30].

The effect of the type and strength of bond between the organic molecule and the particle surface was also investigated by N. Pérez et al. [30] on 5 nm Fe_3−x_O_4_ nanoparticles, synthesized by high-temperature decomposition in organic phase, where oleic acid is covalently bonded to the nanoparticles, in low-temperature aqueous conditions, while polyvinyl alcohol (PVA) yielded just a protective coating without chemical bond. Magnetization and XMCD measurements (Figure 2) showed a saturation magnetization close to bulk magnetite and an orbital moment effectively quenched in oleic acid-coated nanoparticles of high crystal quality. PVA coated nanoparticles showed a reduced value of the magnetization and threefold increase in the orbital moment. The total magnetic moment per formula unit, measured by XMCD, µ_Fe,_ in the oleic acid sample is about 42% larger than that for the PVA sample [µ_Fe_ (PVA) /µ_Fe_ (oleic) = 0.706], in excellent agreement with the magnetization results [M_S_ (PVA) /M_S_ (oleic) = 0.71]. By means of high-resolution electron microscopy measurements, the authors correlated the different nanostructures, arising from different synthesis procedures, to the spin and orbital contribution to the magnetic moments.

The functional group bound to the surface and the type and strength of the bond play a crucial role in tuning magnetic properties. The effect of the oleic acid (OA) on the magnetic anisotropy and saturation magnetization of ~5 nm spinel CoFe_2_O_4_ nanoparticles is compared to that of diethylene glycol (DEG) by Vasilakaki et al. [43] combining magnetization and in field Mössbauer spectroscopy measurements with physical modeling by Density functional theory (DFT) and Monte Carlo simulations. DEG and OA interact with the surface of the particles through hydroxylic (R-OH) and carboxylic (R-COOH) groups, respectively. The difference between the two coatings is in the functional group through which the interaction with the surface occurs: R-OH for DEG and R-COOH for OA, and the type of bond between ligands and the metal atoms, electrostatic for DEG and covalent for OA. 

**Figure 3:**
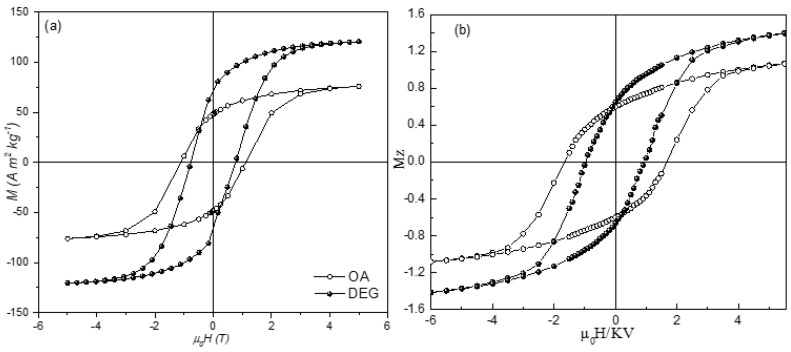
Experimental findings (**a**) and Monte Carlo simulation (**b**) for the hysteresis loops at 5 K for an assembly of interacting CoFe_2_O_4_ nanoparticles coated with DEG (full circles) and OA (empty circles) surfactants. The Figure is reprinted with permission from Vasilakaki et al. [43].

Starting from the cationic distribution, different for the two systems and determined by Mössbauer spectra, the relaxed structures of the nanoparticle systems, anisotropy energy (MAE) and the net magnetic moments were calculated by DFT. The DEG coated sample has a net magnetic moment larger (approximately 1.3 times) and an anisotropy energy smaller (approximately 1.5 times) than those of the OA coated sample. This is coherent with the measured hysteresis cycles at 5 K (Figure 3), showing higher saturation magnetization and lower magnetic anisotropy for the DEG coated sample with respect to the OA one. This provides evidence that the DEG coating is more efficient in reducing the surface disorder. Monte Carlo (MC) simulations, assuming a random distribution of core shell particles and accounting for the interplay between intraparticle surface characteristics and interparticle interactions effect, reproduce very well all the experimental findings.

**Figure 4:**
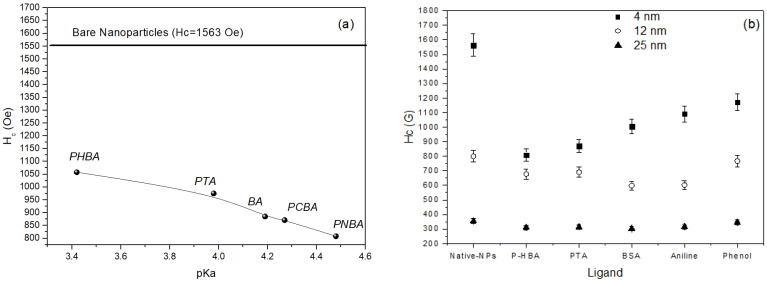
(**a**) variation of coercivity with pKa of MnFe_2_O_4_ coated with different ligands: *p*-hydroxybenzoic acid (PHBA), *p*-toluic acid (PTA), benzoic acid (BA), *p*-chlorobenzoic acid (PCBA), *p*-nitrobenzoic acid (PNBA)). Coercivity of bare nanoparticles is reported as a reference; (**b**) variation of coercivity nanoparticles of different sizes coated with different ligands: *p*-hydroxybenzoic acid (PHBA), *p*-toluic acid (PTA), benzenesulfonic acid (BSA) aniline and phenol.

Beyond the effect of functional group, the chemical nature of the whole ligand represents an important factor in tuning the magnetic properties of nanoparticles. The correlation between the chemical nature of the ligand and the magnetic anisotropy was investigated by C. R. Vestal et al. [24] comparing the effects produced by a series of ligands (para-substituted benzoic acid HOOC–C_6_H_4_–R; R=H, CH_3_, Cl, NO_2_, OH and substituted benzene Y–C_6_H_5_, Y=COOH, SH, NH_2_, OH, SO_3_H) on the coercivity of ~4 nm MnFe_2_O_4_ nanoparticles. The paper makes a very important contribution to the understanding of fundamental properties of surface magnetism, providing evidence of the mechanisms underlying the role of the key factors, closely linked to surface coordination chemistry, responsible for the effects produced by the organic coating. Figure 4a shows the dependence of coercivity on the pKa of the ligands. The coercivity of bare particles is also reported as a reference. The strong decrease of coercivity (almost 50%) observed going from bare to coated particles, can be ascribed to the crystal field splitting energy (CFSE), generated from d orbitals splitting by the coordination ligands, the largest CFSE resulting in the strongest effect, associated to the reduction of spin orbit coupling on the magnetic cations. The acidity of the coordination ligand was found to be the major factor affecting the reduction of the anisotropy. Larger is the pK_a_ (i.e., weaker is the acid, being pKa the negative log of the dissociation constant K_a_), higher is the electron donation capability of the ligand, stronger is the metal-ligand s bond and consequently the CFSE, and larger is the percentage decrease of coercivity at 5 K. Effect of the coating on particles of different size (4, 12, and 25 nm), reported in Figure 4b, indicates a less evident reduction of coercivity in 12 nm particles and practically no effect in 25 nm particles. This is fully consistent with the reduction of the role of the surface, due to the reduced volume fraction of atoms at the surface.

The number of organic molecules attached to the surface and the type of bonding (mono-dentate or bidentate) with the surface atoms significantly affect the magnetic properties of nanoparticles.

Costo et al. [44] investigated the magnetic properties of 3 nm maghemite nanoparticles (synthesized by a gas phase method and recrystallized in acid media) coated by phosphonate (phosphonoacetic acid or pamidronic acid) and carboxylate-based (carboxymethyl dextran) molecules. Magnetization measurements at 5 K showed a decrease of anisotropy (i.e., bare particles: H_c_ = 2130 Oe; pamidronic acid coated particles: H_c_ = 1610 Oe) and an increase of saturation magnetization (i.e., bare particles: M_S_ = 35.2 emu/g; pamidronic acid coated particles: M_S_ = 41.5 emu/g) after both coating processes, due to the reduction of the surface disorder and spin canting. Since the amount of phosphate groups in the sample coated by pamidronic acid is one third approximately of that of the sample coated by phosphonoacetic acid, it is suggested that the first molecules are attached to the particle surface by a double-monodentate bond (similar to what reported by Yee et al. [45] which should facilitate the coordination of ligands inducing a stronger reduction of the surface disorder.

An opposite effect on the saturation magnetization, i.e., a reduction, was reported in references [35,36] where the particles were prepared by methods other than that used by Costo et al. [44]. Ngo et al. [36] investigated the effect of coating on 3 nm cobalt ferrite particles by citric acid. The particles, prepared trough a micellar solution, are characterized by cationic vacancies. A reduction of M_S_ was observed, whereas the anisotropy is the same as for the uncoated particles.

The same effect was observed by Yuan et al. [35] in carboxyl- and amine-functionalized iron oxide nanoparticles, suspended in water, and in oleic acid functionalized suspended in hexane and heptane. A reduction of the magnetic phase was detected depending on the coatings and different suspension media. The largest M_S_ reduction was found for amine coated particles. Oleic acid coated samples showed a solvent-dependent reduction. Besides the difference between the coating molecules, the above results provide evidence of the critical role of the preparation process and coating conditions, affecting the surface characteristics of both the original and coated particles, in determining the magnetic properties of the functionalized particles.

Besides functional groups bound to the nanoparticle surface and chemical nature of the ligands, the chain length of the molecules also plays a role in determining the magnetic properties of the coated particles.

The effects of six organic ligands, characterized by different chain lengths and functional groups (caprylic acid-C8Ac, lauric acid-C12Ac, 1-octanethiol-C8T, 1-dodecanethiol-C12T, 1-octylamine-8Am and 1-dodecylamine -C12Am) on the M_S_ value of 9 nm fcc-FePt nanoparticles were analyzed and compared with the bulk value by Y. Tanaka et al. [46]. They compared the M_S_ values, with respect to the bulk one (75 emu/g), of particles coated by six organic ligands. FePt particles capped with oleic acid (OAc) were synthesized and then the Oac groups were converted to the above ones via ligand exchange. The difference in the M_S_ values was interpreted as the difference in the electron donation ability (basicity) of the ligands to the Fe bands. Regardless of the functional group, the electron donation ability was found to be affected by the chain length of the ligand. Higher M_S_ values were obtained capping the surface of the NPs with ligands with lower basicity (higher pKb) and lower surface coverage, i.e., reducing the number of Fe-ligand binding sites (Table 1). For C8T and C12T samples, the M_S_ values were found to be 7% and 14% higher, respectively, than that of as synthesized NPs capped with oleic acid, due to the thinning of the non-magnetic shell.

**Figure 5:**
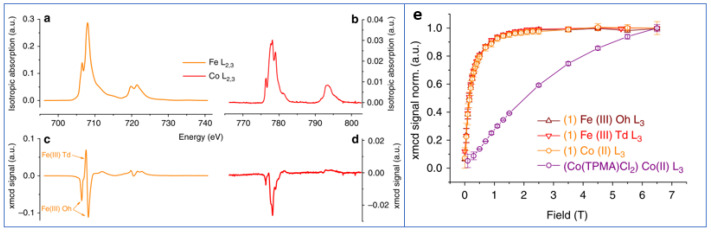
XAS and XMCD signals measured on [Co^II^(TPMA)Cl_2_] functionalized sample at the Fe (**a**,**c**) and Co (**b**,**d**) L_2,3_ edges at 5 K and 6 T. Fe-specific and Co-specific XMCD-detected magnetization curves at 5 K (**e**). The Figure is reprinted with permission from Prado et al. [47].

The surface coating of nanoparticles with molecules exhibiting themselves magnetic properties was found to be a very powerful tool for controlling the magnetic particles. An interesting examples is given by Prado et al. [47] who tuned the magnetic anisotropy of nanoparticles via the surface coordination of anisotropic molecular complexes. They prepared 5 nm γ-Fe_2_O_3_ nanoparticles functionalized by [Co^II^(TPMA)Cl_2_] and TMA (tris (2-pyridylmethyl) amine). A sample functionalized just with TMA was used as a reference. The presence of the {Co^II^(TPMA)}^2+^ complex at the surface of the nanoparticles and the formation of an oxo-bridge between Co(II) and Fe(III) ions were evidenced by XPS, AAS and XAS measurements. The temperature of the maximum in the ZFC curves was higher for [Co_II_(TPMA)Cl^2^] sample (T_max_ = 30 K) than for the reference one (T_max_ = 11 K). Hysteresis loops at 5 K showed a much larger coercivity for the [Co^II^(TPMA)Cl_2_] (H_c_ ~840 Oe) with respect to the sample prepared with addition of Tetramethylammonium hydroxide (H_c_ ~60 Oe). In agreement with the above measurements, ^57^Fe Mössbauer spectra at 77 K indicated a slowdown of the relaxation phenomena of the magnetization, due to the attached Co(II) complexes which increased the magnetic anisotropy of the Fe(III) moments, strengthening thus the magnetization of each nanoparticle. Fe and Co specific (L_2,3_ edges) XMCD-detected magnetization curves at 5 K are superimposed demonstrating that the Co(II) was magnetically coupled to the Fe(III) ions of the maghemite nanoparticles (Figure 5).

## 3. Control of Interparticle Interactions

Besides the effects described above, the coating layer and its thickness allow reducing, through the control of the distance between particle moments, the strength of dipolar interactions [36,37,48,49], which contribute to the effective anisotropy of the nanoparticle assembly. The control of interparticle interactions, which also implies a tuning of the saturation magnetization by the surface coating, is very important for applications, as they usually require concentrated assemblies of magnetic nanoparticles.

Vasilakaki et al. in their paper [43], discussed in the previous section, compared the effect on the magnetic properties of 5 nm CoFe_2_O_4_ nanoparticles produced by the coating with oleic acid (OA) and diethylene glycol (DEG). Lower anisotropy and larger M_S_ (~120 emu/g) were found for the DEG coated sample with respect to the OA coated one (~82 emu/g). For an assembly of single-domain particles with uniaxial anisotropy and magnetization reversal by coherent rotation, the type and strength of interparticle interactions can be estimated using DM plot (ΔM(H) = M_DCD_(H) − (1–2 × M_IRM_(H)) through the Wohlfarth model [50,51]. As shown by the DM plots, the dipolar interactions were found to be stronger (2.5 times) for the DEG-coated sample. This is not due only to the larger M_S_, but also to a shorter interparticle distance between DEG coated particles. In the OA sample, for each particle a ~2 nm thickness of oleic acid produces a total distance of ~4 nm between surfaces. On the other hand, the side chain link of the shorter chain DEG produces a single layer of 0.5 nm with a total distance of 1 nm among particles’ surfaces. The above thicknesses of the OA and the DEG molecules were estimated by a simple calculation starting from density and surface area for 1 g monolayer. The calculated dipolar interactions energies are ~31 K and ~12 K for DEG and OA sample, respectively. The ratio between dipolar energies and DM plot intensities is exactly the same, due to the contribution of the coating to both the saturation magnetization and the interparticle distance. The stronger dipolar interactions in the DEG coated sample results also in a larger blocking temperature with respect to the OA coated sample.

**Figure 6:**
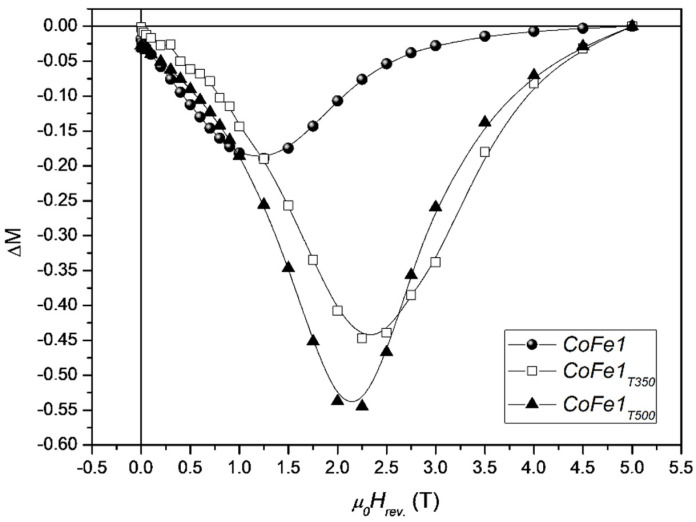
ΔM plot at 5 K for the CoFe1 (full circles), CoFe1T350 (empty squares), and CoFe1T500 (full triangles) samples. The figure is reproduced with permission from ref. [37].

The effect of molecular coating on the interparticle interactions and anisotropy of cobalt ferrite nanoparticles was also investigated by Peddis et al. [37]. Nanoparticles, in powder form, of 5–8 nm average size, were synthesized by the high thermal decomposition method of metalorganic precursor in presence of oleic acid and oleylamine. As shown by X-ray diffraction and TEM images, the cobalt ferrite particles are essentially spherical and uniform, separated from each other by 2 nm surfactant on the surface. Thermal analysis results indicated that the weight percentage of oleic acid is around 30%, corresponding to a monolayer of the organic molecules adsorbed at the surface of the nanoparticles. To evaluate the effect of the coating removal on the interparticle interactions, the 5 nm sample (CoFe1 <DXRD> ~5 nm; M_S_ 70 emu/g; μ_0_Hc ~1.3 T) was submitted to thermal treatments at 350 °C (CoFe1T350, <DXRD> ~5 nm; M_S_ 79 emu/g; μ_0_H_c_ ~1.9 T) and 500 °C (CoFe1T500 <DXRD> ~7.3 nm; M_S_ 72 emu/g; μ_0_H_c_ ~1.8 T). Figure 6 shows ΔM plots for the as prepared cobalt ferrite NPs and after the thermal treatments.

For the three samples, the DM plot shows a negative peak, indicating the predominance of dipolar interparticle interactions. The increase of the DM intensity after thermal treatments at 350 °C can be ascribed mainly to the decomposition of molecular coating (i.e., decreasing of interparticle interactions). In fact, CoFe1 and CoFe1T350 samples, show equal value of particle size, quite close value of M_S_. On the other hand, the treatment at 500 °C induces an increase of particle size and then the further increase of interparticle interactions can be ascribed to the increase of the particle moment (i.e., μ_p_ = MsV). The largest field corresponding to the ΔM peak in the sample without surfactant is a clear indication that the elimination of the molecular coating induces a significant increase of magnetic anisotropy, as confirmed by the increase of coercive field and the effective anisotropy constant.

The effect of the oleate ligands on the interparticle interactions and surface anisotropy of three different capped nanoparticles, Fe_3_O_4_, CoFe_2_O_4_ and ZnFe_2_O_4_ with average size of ∼4.5 nm, was investigated by Virumbrales-del Olmo et al. [52]. The particles were prepared by the high temperature decomposition of metal organic precursor using oleic acid and oleylamine as a stabilizing agent. The coordination of carboxylate groups of the ligands to the surface of the particles was confirmed by FTIR spectroscopy. In the CoFe_2_O_4_ sample, carboxylate groups bridging bidentate act as ligands and in the case of Fe_3_O_4_ the ligands are chelating. However, for ZnFe_2_O_4_ the bridging bidentate and chelating ligands coexist at the surface of the particles. The values of effective magnetic volumes (calculated from the experimental blocking temperature, T_b_, through the expression T_b_ = KV_m_ (1-H/H_k_)^2^/25k_B_, where H and H_k_ are the applied and anisotropy field, respectively), V_eff_, for Fe_3_O_4_ and ZnFe_2_O_4_ samples (71 and 82 nm^3^, respectively) are less than twice the experimental particle volumes, V_TEM_, (61 and 54 nm^3^, respectively) indicating that in these samples 1–2 particles are interacting. However, for the CoFe_2_O_4_ sample, V_eff_ (331 nm^3^) is an order of magnitude higher than V_TEM_ (45 nm^3^), i.e., aggregates of 7–10 nanoparticles. These results suggest that for Fe_3_O_4_ and ZnFe_2_O_4_ nanoparticles the protecting organic layer minimizes the interparticle interactions, while for CoFe_2_O_4_ NPs, despite presenting a similar particle–particle distance (∼2 nm), the molecular coating has less effect on the interparticle interaction. This can be ascribed to the interplay existing to interparticle interactions and magnetic anisotropy, a point that still needs some investigations [48,49].

The control of interparticle interactions, trough the surface coating, can be exploited in some applications improving the performance of nanoparticle based magnetic materials. An interesting example, related to biomedicine, is given by Blanco-Andujar et al. [38]. They investigated the effect of the interparticle interactions, controlled by coating iron oxide NPs by using different concentrations of citric acid (CA), on the intrinsic loss parameter (ILP) for magnetic hyperthermia.

**Figure 7:**
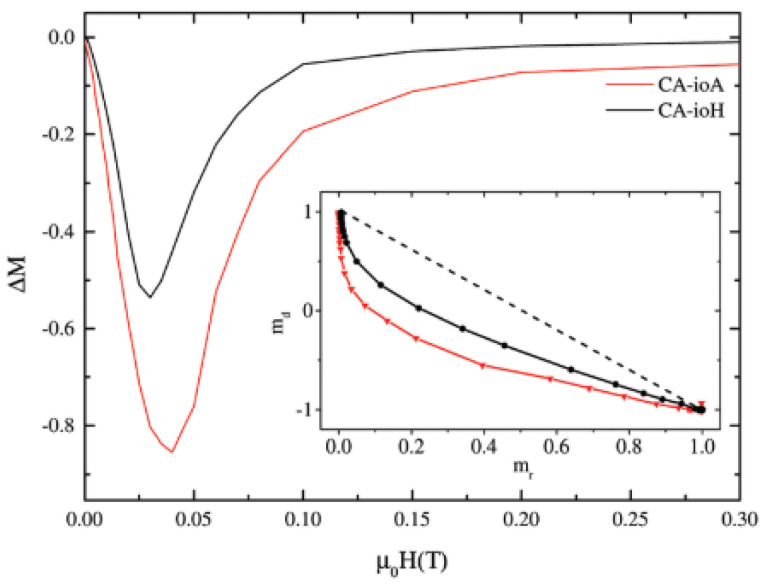
ΔM plots and (inset) Henkel plots of representative’s samples of the CA-io series at 5 K. The figure is reproduced with permission from Blanco-Andujar et al. [38].

A set of citric acid-coated iron oxide NPs was prepared by a coprecipitation method in a microwave reactor. Two NPs assemblies, prepared by adding 1 mmol (CA-ioA) and 2 mmol (CA-ioH) CA in solution, showed the lower (1.8 nHm^2^kg^−1^) and higher (4.1 nHm^2^kg^−1^) ILP values. Room temperature ^57^Fe Mössbauer measurements showed that the samples consist of a mixture of magnetite (25–35 wt%) and maghemite particles. In order to investigate the interparticle interactions, field dependent measurements of the remanent magnetizations (i.e., ΔM plot) have been performed at 5 K (Figure 7). The negative ΔM values indicate that demagnetizing dipole–dipole interactions are dominant. The concentration of citric acid and the reaction time are found to affect the strength of interparticle interactions. In the sample synthesized under lower concentrations of citric acid and longer reaction times (CA-ioA) the interparticle core interactions are stronger. The results provide evidence that the demagnetizing interactions, controlled by the concentration of citric acid, reduce the heating properties.

## 4. Control of Morpho-Structural Properties

The type and the concentration of the organic molecule both during and after the synthesis process can produce significant changes in the morphology of the nanoparticle assembly, allowing to control the particle size and shape, their distribution and their arrangements (i.e., the degree and type of particles aggregation) strongly affecting the magnetic properties [39,40,53,54,55]. Many authors have investigated the effect of the oleic acid concentration on the particle size [40,54,55] and the morphology of nanoparticle assemblies. The effect of the oleic acid concentration on the size, shape, morphology and agglomeration type on the magnetic properties of CoFe_2_O_4_ particles was investigated by Jovanovic et al. [55], who prepared the samples following the same procedure as in [54] using 0.1, 0.15, 0.2, 0.25, 0.5, and 2 M of surfactant.

**Figure 8:**
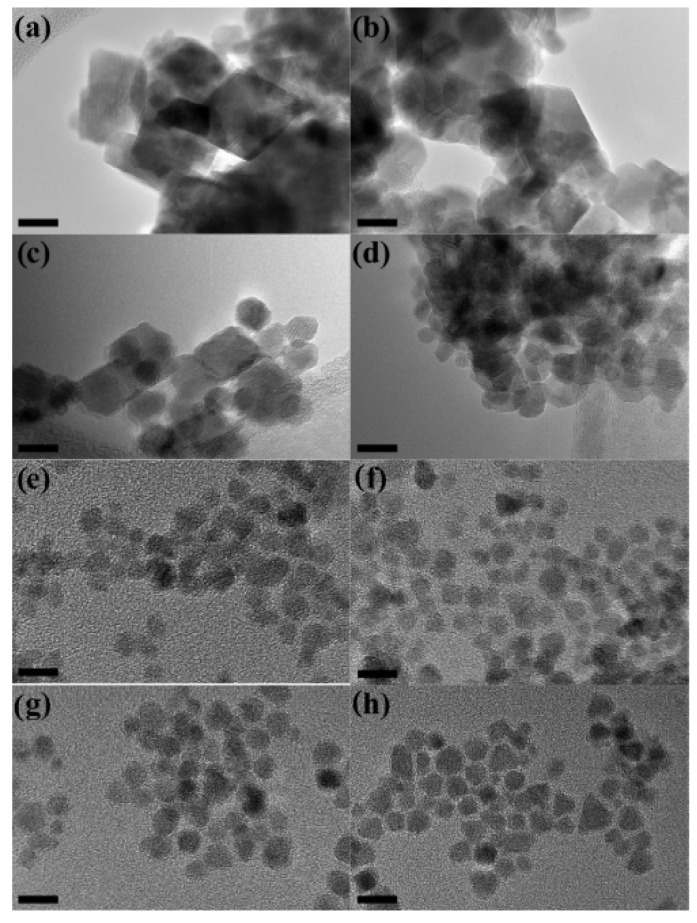
TEM images of cobalt ferrite NPs synthesized with (**a**) 0, (**b**) 0.1, (**c**) 0.15, (**d**) 0.2, (**e**) 0.25, (**f**) 0.5, (**g**) 1, and (**h**) 2 M of oleic acid. Scale bar is 10 nm. The figure is reproduced with permission from Jovanovic et al. [55].

TG and FT-IR analyses showed that oleic acid forms covalent bidentate bonds with metal ions on the particle surface and a complete monolayer at a critical concentration. Without oleic acid, agglomerated nanoplatelets with a crystallite size of about 19 nm were observed by XRD and TEM (Figure 8). A decrease of particles size (from 19 to 5 nm) and change of morphology (from nanoplatelets to sphere-like) was observed increasing the concentration of oleic acid up to 0.25 M, whereas a further increase of the oleic acid concentration had almost no effect on the corresponding properties. Thus, 0.25 M oleic acid can be identified as a critical concentration across which the biggest changes in properties occur. Raman spectroscopy indicated that the surface of the NPs is under strain because of the oleic acid capping layer, while the oleic acid concentration had no effect on the composition and structure of the NPs. Magnetization measurements at room temperature provided evidence that the concentration of OA enables the control of the magnetic behavior of nanoparticles producing a change from ferrimagnetic (below 0.2 M) to superparamagnetic (above 0.25 M).

The effects of coating magnetite nanoparticles (Fe0) by oleic acid (FeOA) and phosphate (FP1) on the morpho-structural features and magnetic properties were investigated and compared by Muthukumaran and Philip [53]. It was observed, by IFTR, that the phosphate capping leads to a nearly cubic shaped particles because of drastic binding of phosphate ions over magnetite crystallites at specific planes as multilayer islands. On the contrary, HRTEM images show that the oleic acid capping produces nearly spherical particles because of slow binding of oleic acid and the homogenous growth of crystal planes in all crystallographic directions. These morphological differences induce significant changes in the magnetic properties, strongly affecting the magnetic stability of the nanoparticles.

**Figure 9:**
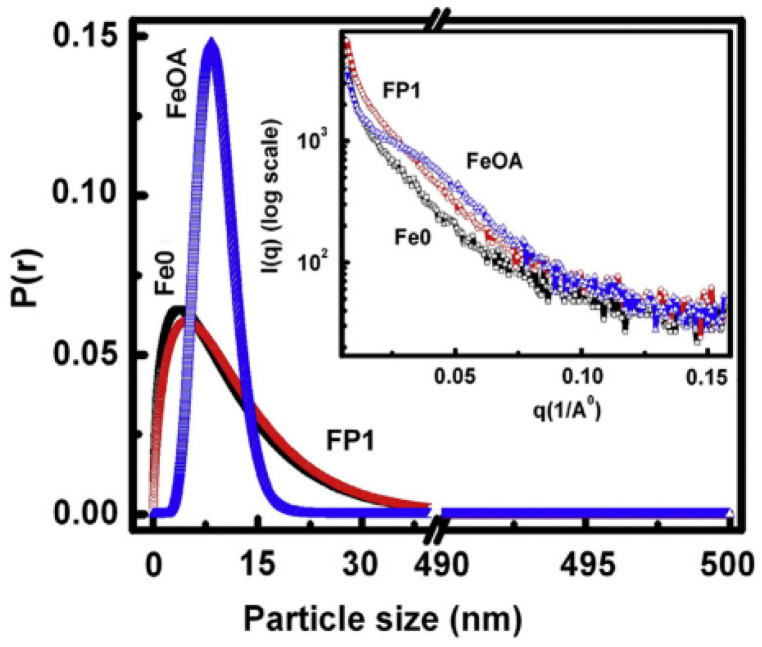
Probability distribution as a function of particle size, obtained from SAXS data, for FP1, FeOA and Fe0 magnetite nanoparticles. The inset shows the scattering intensity vs. q. The figure is reproduced with permission from Muthukumaran et al. [53].

Figure 9 shows the probability distribution of particles, obtained from SAXS data, as a function of their sizes. Almost identical and broad size distribution pattern is observed for the Fe0 and FP1 samples, but both differ widely in comparison to FeOA, exhibiting a very narrow size distribution pattern. This indicates that the interaction of oleic acid with the magnetite NPs was very slow and hence the particles grew sufficiently through ripening of formed nuclei and reached a uniform size with lower defects before arresting the crystallite growth. 

On the other hand, the strong interaction of phosphate ions with the NPs appears to arrest the crystallite growth before attaining a sufficient growth to reach uniform size and shape, which is evident from the identical broad size distribution pattern of FP1 to that of Fe0.

**Figure 10:**
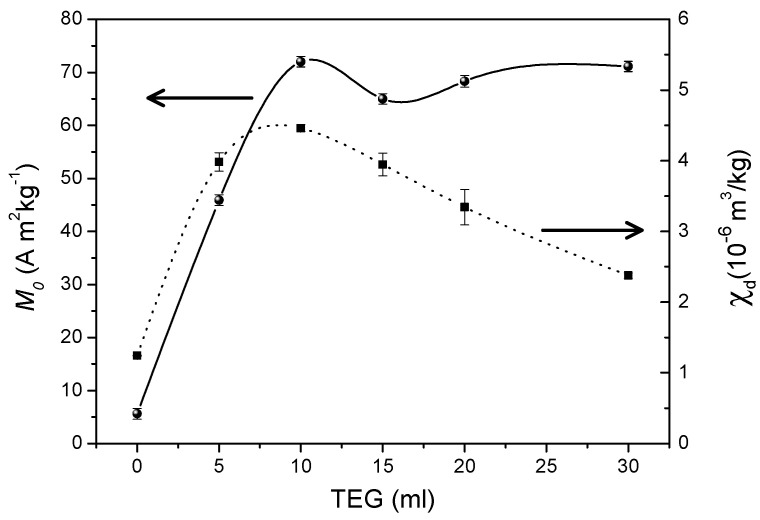
Saturation magnetization (extrapolated at zero field) and high field differential susceptibility (χ_d_) of Mn_2_Fe_2_O_4_ NPs at 5 K with respect to different contents of triethylene glycol.

The effect of the concentration of triethylene glycol on the size of particles aggregates and the saturation magnetization of Mn_2_Fe_2_O_4_ spinel nanoparticles (average size 7.4 nm) was investigated by Aslibeiki et al. [31]. The nanoparticles were synthesized by a solvothermal route based on high temperature decomposition of metal nitrates in the presence of different contents of triethylene glycol (Ml of TEG = 0 (T0), 5 (T5), 10 (T10), 20 (T20), 30 (T30)). Field emission scanning electron microscopy (FE-SEM) showed that with increasing TEG content, the particles agglomeration decreases moving to smaller clusters and enhances the nanoparticles’ polydispersion in the polymer. This leads to a decrease of the blocking temperature, due to the decrease of magnetic interactions between the particle superspins, and to an increasing tendency to saturation, as shown by the field dependence of the magnetization at 300 K (in the superparamagnetic regime) and 5 K (in the blocked regime), and by the high-field differential susceptibility (χ_d_), which was found to decrease by increasing the TEG content, reflecting the tendency to the saturation and the decrease of magnetic disorder at the surface (Figure 10). This is due to O^2-^ ions of the triethylene glycol molecules which bind to Fe and Mn surface cations reducing the surface disorder by decreasing the oxygen vacancies at the NPs surface. This leads to an increase of the magnetization up to a value much higher than that of the bare particles.

The effect of albumin coating on the morphology and magnetic properties of an assembly of ultra-small (∼ 2 nm) MnFe_2_O_4_ nanoparticles was investigated by Vasilakaki et al. [39] by magnetization measurements and numerical simulations at an atomic and mesoscopic scale. The particles were entrapped in bovine serum albumin (BSA) by using a water-in-oil single microemulsion system. It was observed that the covering process induces clustering of particles determining changes in their magnetic properties [56].

**Figure 11:**
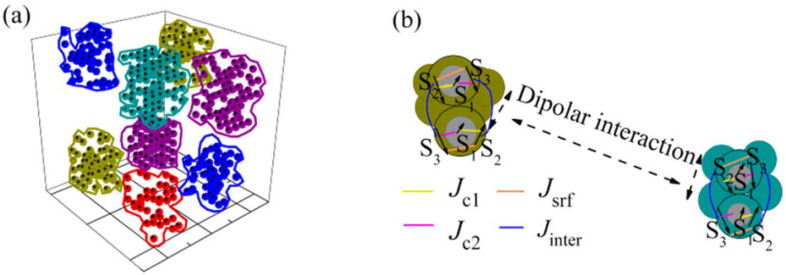
Modeling of the albumin coated clusters of ultra-small MnFe_2_O_4_ nanoparticles with a core/surface morphology (**a**); enlarged schematic representation of two selected dipolarly interacting pairs of nanoparticles, core (S_1_)/surface (S_2_,S_3_), that belong to two neighboring clusters (**b**). In each pair, the exchange intraparticle (J_c1_, J_c2_, J_srf_) and the exchange interparticle interactions (J_inter_) between macrospins are depicted. The figure is reproduced with permission from Vasilakaki et al. [39].

By means of first-principles calculations, based on spin-polarized density functional theory, of the electronic structure, relaxed structures were determined for both the uncoated and albumin coated particles. The clusters are surrounded by the long albumin molecules and then they do not touch each other, whereas the nanoparticles in the clusters are in physical contact (Figure 11). The coating with albumin produces a change in the structure, size and shape distribution of clusters of exchange coupled particles, giving rise to a distribution of blocking temperatures. The calculations showed that the albumin coating reduces the surface anisotropy of the particles leading to a lower value of the coercive field, as experimentally observed.

M.C. simulations, which assumed spherical particles with core/shell randomly located at the nodes of a cubic lattice, accounting for both intra and interparticle contributions to the Hamiltonian, are in very good agreement with the results of experiments.

## 5. Conclusions

The selected papers provide evidence of the key role of the organic coating on the tuning of magnetic properties of nanoparticles. The exploitation of the effects induced by the molecular coating offers a great opportunity to design novel nanostructured magnetic materials (composite and hybrid nanomagnets) opening enormous prospects in the realization of advanced polyfunctional magnetoelectric devices. Moreover, the molecular coating of magnetic nanoparticles, combining inorganic magnetic cores (mainly oxides) and organic shells, plays a very important role in biomedicine (for diagnosis and therapy of tumor cells) not only controlling the anisotropy and increasing the saturation magnetization, making NPs more efficient since they are magnetically driven, but also enhancing their bio-cytocompatibility.

In order to optimize the magnetic properties of nanoparticles for specific applications a deep knowledge of the mechanisms through which the molecule ligands bind to the surface cations is required to understand the role of the many different factors involved (the type of functional group of the ligands, their chain length, their electron donation capability, the nature and the strength of the bond, the thickness of the coating layer, the electronic structure and surface disorder of the original particles, which depend on the synthesis route). The investigations reported in the selected papers provide a contribution to the knowledge of such mechanisms and to a deeper understanding of the surface magnetism effects and their role in controlling the fundamental properties of magnetic nanoparticles. However, the effects of the complex interplay of such factors are not fully clear yet and further investigations are necessary requiring an interdisciplinary approach involving skills in different research fields, such as matter and surface physics, organic and inorganic chemistry and specifically coordination chemistry.

## Figures and Tables

**Table 1:** Saturation magnetization and differential magnetization (with respect to the bulk value, 75 emu/g) of the coated NPs with the ligands.

Ligand	M_S_ (emu/g)	DM_S_ (emu/g)
Oac	19.5	55.5
C8Ac	19.6 ± 0.4	55.4 ± 0.4
C12Ac	15.3 ± 2.8	59.7 ± 2.8
C8T	20.8 ± 0.1	54.2 ± 0.1
C12T	22.3 ± 0.1	52.7 ± 0.1
C8Am	21.0 ± 3.1	54.0 ± 3.1
C12Am	20.4 ± 2.0	54.6 ± 2.0

## Data Availability

Not applicable.

## References

[1] Néel L.M. (1949). Annales de Géophysique. Geophys.

[2] Peddis D., Laureti S., Fiorani D. (2021). New Trends in Nanoparticle Magnetism.

[3] Phan M.H., Alonso J., Khurshid H., Lampen-Kelley P., Chandra S., Repa K.S., Nemati Z., Das R., Iglesias Ó., Srikanth H. (2016). Exchange bias effects in iron oxide-based nanoparticle systems. Nanomaterials.

[4] Déjardin J.L., Vernay F., Kachkachi H. (2020). Specific absorption rate of magnetic nanoparticles: Nonlinear AC susceptibility. J. Appl. Phys..

[5] Vasilakaki M., Gemenetzi F., Devlin E., Yi D.K., Riduan S.N., Lee S.S., Ying J.Y., Papaefthymiou G.C., Trohidou K.N. (2021). Size effects on the magnetic behavior of γ-Fe_2_O_3_ core/SiO_2_ shell nanoparticle assemblies. J. Magn. Magn. Mater..

[6] Roldan M.A., Mayence A., López-Ortega A., Ishikawa R., Salafranca J., Estrader M., Salazar-Alvarez G., Dolors Baró M., Nogués J., Pennycook S.J. (2021). Probing the meta-stability of oxide core/shell nanoparticle systems at atomic resolution. Chem. Eng. J..

[7] Sánchez E.H., Vasilakaki M., Lee S.S., Normile P.S., Muscas G., Murgia M., Andersson M.S., Singh G., Mathieu R., Nordblad P. (2020). Simultaneous individual and dipolar collective properties in binary assemblies of magnetic nanoparticles. Chem. Mater..

[8] Rizzuti A., Dassisti M., Mastrorilli P., Sportelli M.C., Cioffi N., Picca R.A., Agostinelli E., Varvaro G., Caliandro R. (2015). Shape-control by microwave-assisted hydrothermal method for the synthesis of magnetite nanoparticles using organic additives. J. Nanopart. Res..

[9] Varvaro G., Imperatori P., Laureti S., Cannas C., Ardu A., Plescia P., Capobianchi A. (2020). Synthesis of L10 alloy nanoparticles. Potential and versatility of the pre-ordered Precursor Reduction strategy. J. Alloys Compd..

[10] Bárcena C., Sra A.K., Gao J. (2009). Applications of magnetic nanoparticles in biomedicine. Nanoscale Magnetic Materials and Applications.

[11] Gupta A.K., Gupta M. (2005). Synthesis and surface engineering of iron oxide nanoparticles for biomedical applications. Biomaterials.

[12] Scialabba C., Puleio R., Peddis D., Varvaro G., Calandra P., Cassata G., Cicero L., Licciardi M., Giammona G. (2017). Folate targeted coated SPIONs as efficient tool for MRI. Nano Res..

[13] Curcio M., Rau J.V., Santagata A., Teghil R., Laureti S., De Bonis A. (2019). Laser synthesis of iron nanoparticle for Fe doped hydroxyapatite coatings. Mater. Chem. Phys..

[14] Scherer C., Figueiredo Neto A.M. (2005). Ferrofluids: Properties and applications. Braz. J. Phys..

[15] Rossi L.M., Costa N.J.S., Silva F.P., Wojcieszak R. (2014). Magnetic nanomaterials in catalysis: Advanced catalysts for magnetic separation and beyond. Green Chem..

[16] Zhang Q., Yang X., Guan J. (2019). Applications of Magnetic Nanomaterials in Heterogeneous Catalysis. ACS Appl. Nano Mater..

[17] Sobczak-Kupiec A., Venkatesan J., Alhathal AlAnezi A., Walczyk D., Farooqi A., Malina D., Hosseini S.H., Tyliszczak B. (2016). Magnetic nanomaterials and sensors for biological detection. Nanomed. Nanotechnol. Biol. Med..

[18] Nejad F.G., Tajik S., Beitollahi H., Sheikhshoaie I. (2021). Magnetic nanomaterials based electrochemical (bio)sensors for food analysis. Talanta.

[19] Tuantranont A. (2013). Applications of Nanomaterials in Sensors and Diagnostics.

[20] Frey N.A., Peng S., Cheng K., Sun S. (2009). Magnetic nanoparticles: Synthesis, functionalization, and applications in bioimaging and magnetic energy storage. Chem. Soc. Rev..

[21] Zhang H.W., Liu Y., Sun S. (2010). heng Synthesis and assembly of magnetic nanoparticles for information and energy storage applications. Front. Phys. China.

[22] Chiba D., Yamanouchi H., Hatsukura F., Ohno H. (2003). Electrical manipulation of magnetization reversal in a ferromagnetic semiconductor. Science.

[23] Néel L.M. (1954). Anisotropie magnétique superficielle et surstructures d’orientation Al. G. Phys. Radium.

[24] Vestal C.R., Zhang Z.J. (2003). Effects of surface coordination chemistry on the magnetic properties of MnFe_2_O_4_ spinel ferrite nanoparticles. J. Am. Chem. Soc..

[25] Gradmann U. (1991). Surface magnetism. J. Magn. Magn. Mater..

[26] Fiorani D. (2005). Surface Effects in Magnetic Nanoparticles.

[27] Laureti S., Gerardino A., D’Acapito F., Peddis D., Varvaro G. (2021). The role of chemical and microstructural inhomogeneities on interface magnetism. Nanotechnology.

[28] Nogués J., Schuller I.K. (1999). Exchange bias. J. Magn. Magn. Mater..

[29] Berkowitz A.E., Takano K. (1999). Exchange anisotropy—A review. J. Magn. Magn. Mater..

[30] Ṕrez N., Bartoloḿ F., García L.M., Bartoloḿ J., Morales M.P., Serna C.J., Labarta A., Batlle X. (2009). Nanostructural origin of the spin and orbital contribution to the magnetic moment in Fe_3-x_O_4_ magnetite nanoparticles. Appl. Phys. Lett..

[31] Aslibeiki B., Kameli P., Ehsani M.H., Salamati H., Muscas G., Agostinelli E., Foglietti V., Casciardi S., Peddis D. (2016). Solvothermal synthesis of MnFe_2_O_4_ nanoparticles: The role of polymer coating on morphology and magnetic properties. J. Magn. Magn. Mater..

[32] Nagesha D.K., Plouffe B.D., Phan M., Lewis L.H., Sridhar S., Murthy S.K. (2009). Functionalization-induced improvement in magnetic properties of Fe3 O4 nanoparticles for biomedical applications. J. Appl. Phys..

[33] Salafranca J., Gazquez J., Pérez N., Labarta A., Pantelides S.T., Pennycook S.J., Batlle X., Varela M. (2012). Surfactant organic molecules restore magnetism in metal-oxide nanoparticle surfaces. Nano Lett..

[34] Mirzaee S., Farjami Shayesteh S., Mahdavifar S. (2014). Anisotropy investigation of cobalt ferrite nanoparticles embedded in polyvinyl alcohol matrix: A Monte Carlo study. Polymer (Guildf).

[35] Yuan Y., Rende D., Altan C.L., Bucak S., Ozisik R., Borca-Tasciuc D.A. (2012). Effect of surface modification on magnetization of iron oxide nanoparticle colloids. Langmuir.

[36] Ngo A.T., Bonville P., Pileni M.P. (1999). Nanoparticles of CoxFey□zO4: Synthesis and superparamagnetic properties. Eur. Phys. J. B.

[37] Peddis D., Orrù F., Ardu A., Cannas C., Musinu A., Piccaluga G. (2012). Interparticle Interactions and Magnetic Anisotropy in Cobalt Ferrite Nanoparticles: Influence of Molecular Coating. Chem. Mater..

[38] Blanco-Andujar C., Ortega D., Southern P., Pankhurst Q.A., Thanh N.T.K. (2015). High performance multi-core iron oxide nanoparticles for magnetic hyperthermia: Microwave synthesis, and the role of core-to-core interactions. Nanoscale.

[39] Vasilakaki M., Ntallis N., Bellusci M., Varsano F., Mathieu R., Fiorani D., Peddis D., Trohidou K.N. (2020). Effect of albumin mediated clustering on the magnetic behavior of MnFe_2_O_4_ nanoparticles: Experimental and theoretical modeling study. Nanotechnology.

[40] Herojit Singh L., Govindaraj R., Amarendra G., Sundar C.S. (2013). Atomic scale study on the thermal evolution of local structure and magnetic properties in oleic acid coated iron oxide nanoparticles. J. Phys. Chem. C.

[41] Zhang D.E., Zhang X.J., Ni X.M., Zheng H.G., Yang D.D. (2005). Synthesis and characterization of NiFe_2_O_4_ magnetic nanorods via a PEG-assisted route. J. Magn. Magn. Mater..

[42] Pettersson L.G.M., Nilsson A. (2014). A molecular perspective on the d-band model: Synergy between experiment and theory. Top. Catal..

[43] Vasilakaki M., Ntallis N., Yaacoub N., Muscas G., Peddis D., Trohidou K.N. (2018). Optimising the magnetic performance of Co ferrite nanoparticles: Via organic ligand capping. Nanoscale.

[44] Costo R., Morales M.P., Veintemillas-Verdaguer S. (2015). Improving magnetic properties of ultrasmall magnetic nanoparticles by biocompatible coatings. J. Appl. Phys..

[45] Yee C., Kataby G., Ulman A., Prozorov T., White H., King A., Rafailovich M., Sokolov J., Gedanken A. (1999). Self-assembled monolayers of alkanesulfonic and -phosphonic acids on amorphous iron oxide nanoparticles. Langmuir.

[46] Tanaka Y., Saita S., Maenosono S. (2008). Influence of surface ligands on saturation magnetization of FePt nanoparticles. Appl. Phys. Lett..

[47] Prado Y., Daffé N., Michel A., Georgelin T., Yaacoub N., Grenèche J.M., Choueikani F., Otero E., Ohresser P., Arrio M.A. (2015). Enhancing the magnetic anisotropy of maghemite nanoparticles via the surface coordination of molecular complexes. Nat. Commun..

[48] Muscas G., Peddis D., Cobianchi M., Lascialfari A., Cannas C., Musinu A., Omelyanchik A., Rodionova V., Fiorani D., Mameli V. (2019). Magnetic interactions vs. magnetic anisotropy in spinel ferrite nanoparticles. IEEE Magn. Lett..

[49] Muscas G., Concas G., Laureti S., Testa A.M., Mathieu R., De Toro J.A., Cannas C., Musinu A., Novak M.A., Sangregorio C. (2018). Interplay between single particle anisotropy and interparticle interactions in ensembles of magnetic nanoparticles. Phys. Chem. Chem. Phys..

[50] Wohlfarth E.P. (1958). Relations between different modes of acquisition of the remanent magnetization of ferromagnetic particles. J. Appl. Phys..

[51] Kelly P.E., O’Grady K., Mayo P.L., Chantrell R.W. (1989). Switching mechanisms in cobalt-phosphorus thin films. IEEE Trans. Magn..

[52] Virumbrales-Del Olmo M., Delgado-Cabello A., Andrada-Chacón A., Sánchez-Benítez J., Urones-Garrote E., Blanco-Gutiérrez V., Torralvo M.J., Sáez-Puche R. (2017). Effect of composition and coating on the interparticle interactions and magnetic hardness of MFe_2_O_4_ (M = Fe, Co, Zn) nanoparticles. Phys. Chem. Chem. Phys..

[53] Muthukumaran T., Philip J. (2016). Effect of phosphate and oleic acid capping on structure, magnetic properties and thermal stability of iron oxide nanoparticles. J. Alloys Compd..

[54] Ansari S.M., Sinha B.B., Phase D., Sen D., Sastry P.U., Kolekar Y.D., Ramana C.V. (2019). Particle Size, Morphology, and Chemical Composition Controlled CoFe_2_O_4_ Nanoparticles with Tunable Magnetic Properties via Oleic Acid Based Solvothermal Synthesis for Application in Electronic Devices. ACS Appl. Nano Mater..

[55] Jovanović S., Spreitzer M., Tramšek M., Trontelj Z., Suvorov D. (2014). Effect of oleic acid concentration on the physicochemical properties of cobalt ferrite nanoparticles. J. Phys. Chem. C.

[56] Bellusci M., La Barbera A., Seralessandri L., Padella F., Piozzi A., Varsano F. (2009). Preparation of albumin-ferrite superparamagnetic nanoparticles using reverse micelles. Polym. Int..

